# Hot-Melt 3D Extrusion for the Fabrication of Customizable Modified-Release Solid Dosage Forms

**DOI:** 10.3390/pharmaceutics12080738

**Published:** 2020-08-05

**Authors:** Jaemin Lee, Chanwoo Song, Inhwan Noh, Sangbyeong Song, Yun-Seok Rhee

**Affiliations:** College of Pharmacy and Research Institute of Pharmaceutical Sciences, Gyeongsang National University, Jinju 52828, Korea; applemast@gnu.ac.kr (J.L.); cwsong@gnu.ac.kr (C.S.); oinhoan323@gnu.ac.kr (I.N.); songsang59@gnu.ac.kr (S.S.)

**Keywords:** hot-melt extrusion, 3D printing, controlled release, immediate release, pharmaceutical manufacturing, personalized medication, ibuprofen, solid dispersion, solubilization, polyvinyl pyrrolidone

## Abstract

In this work, modified-release solid dosage forms were fabricated by adjusting geometrical properties of solid dosage forms through hot-melt 3D extrusion (3D HME). Using a 3D printer with air pressure driving HME system, solid dosage forms containing ibuprofen (IBF), polyvinyl pyrrolidone (PVP), and polyethylene glycol (PEG) were printed by simultaneous HME and 3D deposition. Printed solid dosage forms were evaluated for their physicochemical properties, dissolution rates, and floatable behavior. Results revealed that IBF content in the solid dosage form could be individualized by adjusting the volume of solid dosage form. IBF was dispersed as amorphous state with enhanced solubility and dissolution rate in a polymer solid dosage form matrix. Due to absence of a disintegrant, sustained release of IBF from printed solid dosage forms was observed in phosphate buffer at pH 6.8. The dissolution rate of IBF was dependent on geometric properties of the solid dosage form. The dissolution rate of IBF could be modified by merging two different geometries into one solid dosage form. In this study, the 3D HME process showed high reproducibility and accuracy for preparing dosage forms. API dosage and release profile were found to be customizable by modifying or combining 3D modeling.

## 1. Introduction

Personalized medication has received attention from the medical society because of different drug effects caused by genetic background [1]. Current pharmaceutical manufacturing is based on mass production which produces pharmaceutical dosage forms with small variations in doses and API release profiles [2]. Because of such low flexibility, there are few options to adjust doses for individualized pharmacotherapy such as dosing liquid and dividing dosage forms into small units. However, these options are usually not suitable for controlled-release dosage forms and can lead high inaccuracy of dose uniformity [3]. As a result, the need for a new method for preparing personalized medication has increased. Three-dimensional (3D) printing technology has received attention as a novel candidate for future pharmaceutical manufacturing because of its advantages such as on-demand manufacturing, ability to fabricate complex structure, high accuracy, reproducibility, and cost effectiveness [4].

Pharmaceutical applications of various types of 3D printer such as digital light processing [5], inkjet printing [6], selective laser sintering [7], semi-solid extrusion [8], and fused deposition modeling (FDM) have been studied for years. FDM has been reported to be useful for preparing solid dispersion of active pharmaceutical ingredients (APIs) [9]. FDM is a printing technology based on layer-by-layer deposition of molten or softened materials from the printer head [10]. FDM is considered to be highly cost efficient, easily accessible, and applicable for preparation of model of complex geometries with good mechanical properties. Several studies have applied FDM for preparation of various pharmaceutical dosage forms such as extended-release tablet [11], floating tablet [12], orally disintegrating film [13] and suppository [14]. FDM printer with dual nozzle can extrude two different filament for preparation of composite tablet [15]. FDM 3D printer usually requires thermoplastic materials in the form of filament for feeding to the printer head. Filament needs to have appropriate mechanical properties, flexibility, thermal stability and melt viscosity for successful 3D printing process [16]. It has been reported that hot-melt extrusion (HME) is useful for preparing filament to facilitate consecutive FDM [17,18]. Nevertheless, the pre-process step required for preparing filament remains as a limitation [19,20].

By using hot-melt 3D extrusion, it is possible to deposit molten or soften materials in an on-demand process without requiring an additional pre-process step for preparing filament. Despite this advantage, reports on pharmaceutical applications of a hot-melt extrusion 3D printer are limited. Application of HME 3D printer for preparation of modified-release dosage forms remains unexplored. This work demonstrates pharmaceutical application of HME 3D printer for preparing solid dosage forms with various dissolution profiles. To investigate the effect of geometrical properties on API release profiles, solid dosage forms with various shapes and sizes were prepared. Solid dosage forms were fused together in various combinations to modify the dissolution rate of API. The effect of thermodynamic condition of printing process on solid-state of API was investigated. Physicochemical properties, dissolution rates, and floatable behavior of printed solid dosage forms were also evaluated.

## 2. Materials and Methods

### 2.1. Materials

PVP Kollidon 12 PF (PVP K12) was gifted by BASF Korea (Seoul, Korea). PEG 1500 was purchased from Daejung Chemicals and Metals (Siheung, Korea). Ibuprofen was gifted by KyungDong Pharm (Seoul, Korea). Ibuprofen reference, sodium hydroxide, potassium phosphate monobasic, and sodium chloride were purchased from Sigma-Aldrich (St. Louis, MO, USA). Hydrochloric acid solution was purchased from Merck Millipore (Burlington, MA, USA). All other materials used were of high quality (HPLC grade or better). Plate form of PEG 1500 was pulverized with a mortar and then sieved with #20 sieve before use.

### 2.2. Formulation and Printing Process

Solid dosage form consisted of PEG 1500 as a plasticizer, PVP K12 as a binder and diluent, and ibuprofen as a model API at a ratio of 5:75:20. Ibuprofen has a melting point near 77 °C. Because of the high temperature condition of hot-melt extrusion 3D printing process, ibuprofen undergoes a melting event. It is then mixed with other excipients. Incorporation of small molecule into polymer structure can enhance the flowability of molten mixture [21]. Moreover, ibuprofen can be utilized as a nontraditional plasticizer for extrusion of ethyl cellulose [22]. HME 3D printer uses small diameter of nozzle for high resolution. Because of the plasticizing effect for preventing printing problems such as nozzle clogging and irregular amount of extrusion, ibuprofen was selected as a model API. As a polymer, PVP K12 undergoes glass transition at temperature above 90 °C. Because of such thermoplastic property, the higher the temperature, the lower the viscosity. The flowability of the molten mixture should be high enough to prevent printing problems. PVP K12 has relatively low molecular weight and glass transition temperature than PVP K17, PVP K30, and PVP K90. PVP K12 also shows low melt viscosity under 1000 Pa·s at 160 °C [23]. The relatively low melt viscosity can prevent nozzle clogging during printing process. Moreover, PVP K12 has higher degradation temperature (225 °C) than that of PVP K17 (175 °C), PVP K30 (175 °C) and PVP K90 (200 °C). Melting point of PEG 1500 is near 46 °C. Due to difference in extrusion mechanism between hot-melt air-extrusion 3D printing and usual HME system which uses screw to push material out, the flowability of molten mixture and miscibility to each component are essential to prevent printing problems of phase separation, dose uniformity, resolution of modeling, and nozzle clogging. PEG 1500 as a plasticizer was incorporated into a polymeric matrix [24].

**Figure 1:**
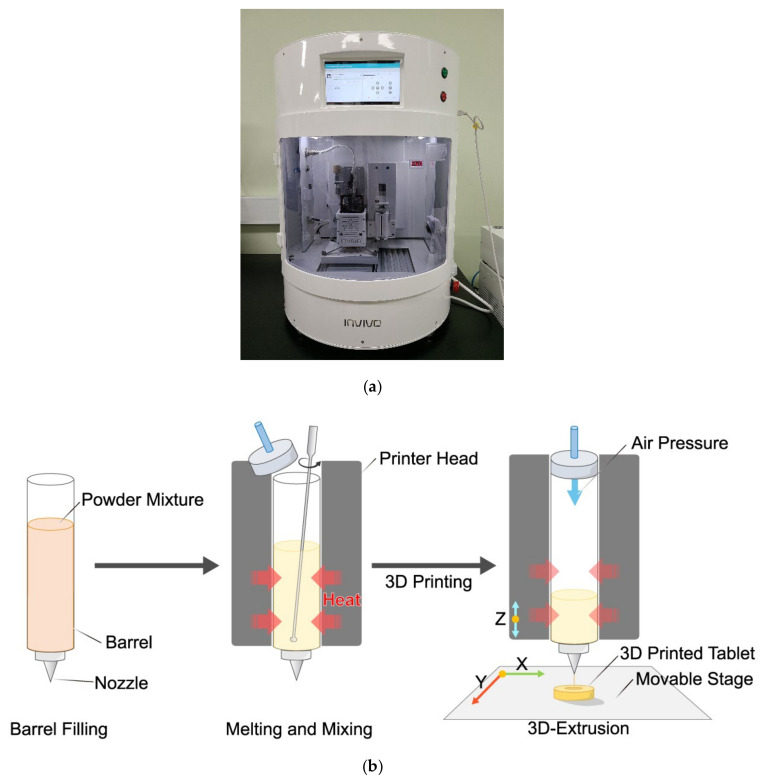
(**a**) ROKIT INVIVO Premium hot-melt air-extrusion 3D printer and (**b**) Schematic diagram of hot-melt 3D extrusion.

An air pressure driving HME based 3D printer (ROKIT INVIVO Premium, ROKIT healthcare, Seoul, Korea) was used in this study. Figure 1 shows process of hot-melt 3D extrusion. 3D models of solid dosage forms were designed with Rhinoceros software version 5 (Robert McNeel & Associates, Seattle, WA, USA), exported as stereolithography file (.stl) format, and imported into 3D printer software (NewCreatorK, ROKIT healthcare, Seoul, Korea) for setting printing parameters. Printing speed was set at 12 mm/s. Diameter of nozzle was 0.2 mm. Layer thickness was set at 0.4 mm. Infill rate was 50%. Temperatures of barrel and bed were 155 °C and 25 °C, respectively. After setting printing parameters, generated GCODE file was uploaded to the 3D printer.

To have a stable printing process, the flow rate of the molten mixture through nozzle needs to be high enough. If not, nozzle clogging can happen. Therefore, we set the air pressure to be 125 kPa. Because of the high flowability and such a high air pressure, the amount of extrusion needed to be controlled because excessive amount of extrusion might cause collapse of the model structure and damage the nozzle. By adjusting the printing speed to be 12 mm/s, the thickness of extrusion was reduced and the resolution was enhanced.

Powder mixture (5 g) of PEG/PVP/Ibuprofen (5/75/20) was poured into a barrel. The barrel was then heated to 155 °C which was much higher than the glass transition temperature of PVP K12. Molten mixture was then mixed with a spatula during heating. Nozzle was pre-heated before printing above 10 °C than the printing temperature to prevent nozzle clogging. Pre-heated nozzle was then mounted to the barrel right before printing. After nozzle mounting, printing was started according to the imported GCODE file.

### 2.3. Dissolution Test

Dissolution test performed by Jain et al. [25] and Rivera-Leyva et al. [26] was adapted with slight modification. Dissolution test was performed using USP-II apparatus (DT 126 lite, Erweka, Langen, Germany). Six solid dosage forms were introduced to the dissolution vessel which was filled with 900 mL of HCl solution (pH 1.2) or potassium phosphate solution (pH 6.8). The temperature was maintained at 37 ± 0.5 °C with stirring at 50 rpm. At fixed time points, 5 mL of sample solution was collected manually and the same volume of test solution was added. Acquired samples were filtered using 0.2 μm syringe filters (Econofilter, Agilent Technologies, Santa Clara, CA, USA) and diluted with initial condition of HPLC mobile phase.

The % dissolution efficiency (% DE) was calculated to compare the relative effect of geometrical properties of solid dosage forms. The % DE of pharmaceutical dosage form is defined as the area under the dissolution curve up to a certain time, t, express as a percentage of the area of the rectangle described by 100% dissolution at the same time. [27] The % DE can be calculated as follows:

(1) $$\%\;\mathrm{DE}=\frac{\int_{0}^{t}y\;\mathrm{dt}}{y_{100}\times t}\times 100$$

where, y is the % cumulative release of API at time t.

### 2.4. API Content Test

Six solid dosage forms were dissolved in 50% methanol aqueous solution. The final volume of the solution was set to be 50 mL in a volumetric flask. API solution was diluted 100 times with initial condition of HPLC mobile phase. All samples were filter with 0.2 μm syringe filters (Econofilter, Agilent Technologies, Santa Clara, CA, USA). API content was determined by HPLC.

### 2.5. Chromatographic Condition

Quantification of ibuprofen was performed with an Agilent 1260 series HPLC system (Agilent Technologies, Santa Clara, CA, USA). Chromatographic separation of samples was carried out with Kinetex 5µ EVO C18 100 Å columns (250 × 4.6 mm, Phenomenex, Torrance, CA, USA). The mobile phase consisted of 10 mM ammonium formate (pH 3.5) aqueous solution (A) and acetonitrile (B). A gradient program with a flow rate of 1.0 mL/min was applied while maintaining the column temperature at 30 °C. The initial composition of mobile phase was 30% of B which was maintained for 1 min. The ratio of B was then increased linearly from 30% to 70% for 5 min and maintained for 5 min. The gradient was then returned to the initial condition for 2.5 min and kept for 5.5 min. The total analysis time was 19 min for each sample. The injection volume was 20 μL and UV absorbance was detected at 222 nm. The retention time of ibuprofen was near 8.6 min. The calibration curve was linear at concentration range of 1–50 μg/mL (*R*^2^ > 0.9999, *n* = 6).

### 2.6. Physical Parameters Measurement

The true density of printed solid dosage form was measured in grams per cubic centimeters by helium pycnometer using an AccuPyc 1340 helium pycnometer (Micrometrics instruments Co., Norcross, GA, USA). Apparent volume (*V*) of cylinder dosage form was measured with Vernier calipers (Mitutoyo, Kawasaki, Japan). The solid fraction (*SF*) and porosity (ε) of cylinder dosage form were calculated based on the true density (*ρ_true_*), apparent volume (*V*), and weight (*Wt*) as below [28]:

(2) $$SF=\frac{Wt}{\rho_{true}\times V}$$

(3) ε=1−SF

Physical parameters of solid dosage forms such as apparent volume (*V*), surface area (SA), and apparent density were calculated using true density, true volume, and porosity. The same porosity (11.3% ± 0.2) was applied to the calculations because the true density (1.160 g/cm^3^ ± 0.004) measured for all solid dosage forms was almost the same.

### 2.7. Floating Test

Floating test method performed by Huanbutta et al. [29] was adapted with slight modification. Floating duration time was measured for floatable solid dosage forms using a shaker (Rotamax 120, Heidolph Instruments, Schwabach, Germany). Each sample was added to 400 mL of water in a beaker which was shaken at 100 RPM to inhibit the adhesion of to the beaker.

### 2.8. X-Ray Diffraction

For solid-state characterization, X-ray diffractometer D8 Advance A25 (BRUKER Co., Billerica, USA) with Cu Kα radiation (λ = 1.5406 Å) was used to obtain X-ray diffractograms of raw powders of ibuprofen, PVP k12, PEG 1500, physical mixture, and extrudates. Voltage and current of X-ray beam were set at 40 kV and 40 mA, respectively. Angular scan range was 5° < 2*θ* < 60° using a continuous scan mode.

### 2.9. Surface Morphology

Surface morphologies of PVP K12, physical mixture, and extrudate were analyzed using a SEM (JSM-6380 LV, JEOL, Tokyo, Japan) at 15 kV. All samples were coated with gold under vacuum for 120 s prior to observation using an ion coater SPT-20 (Coxem Co., Daejeon, Korea).

### 2.10. Thermal Analysis

Thermogravimetry analysis (TGA) and differential thermal analysis (DTA) were performed to characterize solid-state and thermal stability of materials using a Q600 (TA Instruments, New Castle, DE, USA). Samples were heated on aluminum pans from ambient temperature to 400 °C at a heating rate of 10 °C/min with constant nitrogen flow at a rate of 100 mL/min.

### 2.11. Statistical Analysis

All data are presented as the mean ± standard deviation (SD) of at least three separate experiments. Data were compared using the Student *t*-test and *p* values less than 0.05 were considered statistically significant.

## 3. Results

### 3.1. Physical Properties

**Figure 2:**
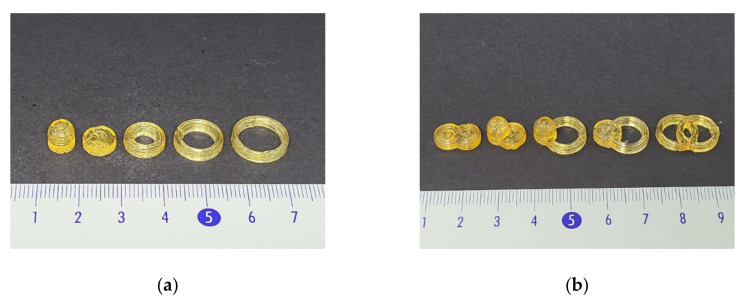
(**a**) Appearance of printed solid dosage forms of float, cylinder, D10 ring, D12 ring, and D14 ring from the left. (**b**) Appearance of printed combined solid dosage forms of CC, CF, RF, CR, and RR from the left.

Figure 2 shows the appearance of printed solid dosage forms. Solid dosage forms showed yellowish and transparent surface of matrix. Partial coalescence of each layer was observed. It might be caused by slow solidification after extrusion due to a high temperature. However, there was no significant collapse of structure. Printed solid dosage forms showed high similarity with 3D modeling. Solid dosage forms with different shapes such as cylinder, ring, and float solid dosage form with cavity inside for floatability were printed to evaluate the effect of geometrical properties on dissolution profile. In the case of ring solid dosage form, two additional ring solid dosage forms with different diameters (12 mm (D12) and 14 mm (D14)) and thickness were printed to better investigate effects of geometrical properties such as surface area and volume. A total of five single solid dosage forms were prepared. Fusion of two solid dosage forms in various combination of cylinder, D12 ring, and float were performed to modify API release profiles. Solid dosage forms were fused to have five different combinations: Cylinder–cylinder (CC), cylinder–float (CF), ring–float (RF), cylinder–ring (CR), and ring–ring (RR).

Table 1 shows physical properties of printed solid dosage forms. To better investigate the geometrical effect, surface area/volume (SA/V) was calculated. In the case of single solid dosage form, float solid dosage form had the smallest SA/V value while D14 ring had the highest SA/V value (D14 > D12 > D10 > cylinder > float). Geometrical properties of solid dosage forms were adjusted by fusion of solid dosage forms depending on physical characteristics of applied solid dosage form compartment. The SA/V value of CC was increased by compartment replacement of cylinder to ring. On the other hand, addition of float compartment decreased the SA/V ratio.

### 3.2. Solid-State of API

**Figure 3:**
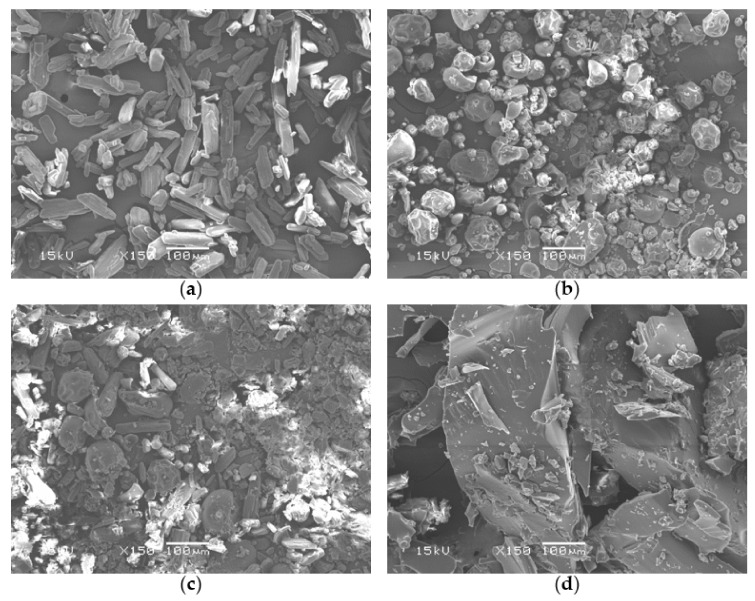
SEM images of (**a**) raw powder of ibuprofen, (**b**) PVP Kollidon 12 PF (PVP K12), (**c**) physical mixture, and (**d**) extrudate.

As shown in Figure 3, SEM images revealed that ibuprofen powder comprised long and needle-like crystals with a smooth surface. Ibuprofen crystals and PVP particles were observed partially in SEM images of physical mixture. Extrudate particles showed a glassy surface and an amorphous shape. Significant deformation of particles of component was observed in SEM images of extrudate. There were no significant traces of crystalline form of ibuprofen in extrudate.

**Figure 4:**
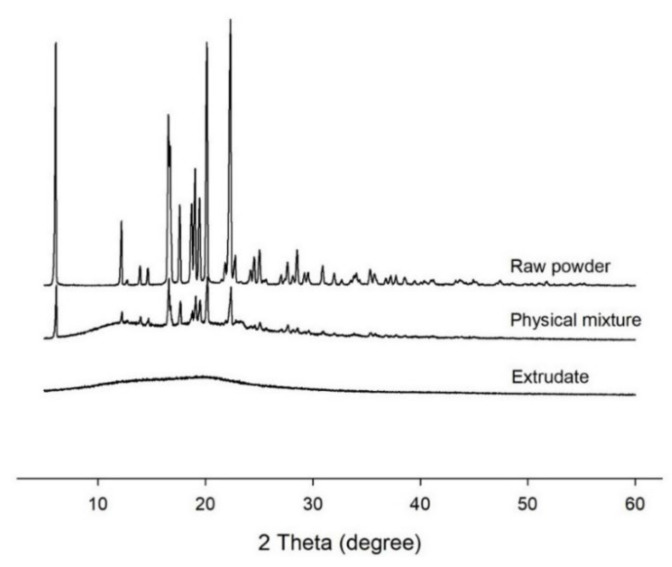
X-ray diffractogram of materials of raw active pharmaceutical ingredients (API) powder, physical mixture, and extrudate.

Diffractograms of ibuprofen powder, PVP K12, physical mixture, and extrudate at diffraction angle of 2θ are shown in Figure 4. As shown in Figure 4, distinctive peaks of ibuprofen were observed at 6.084, 16.556, 20.1353, and 22.323, indicating the crystalline nature of the API. Only physical mixture showed the same characteristic peaks as ibuprofen, indicating the presence of crystalline form of ibuprofen. No significant peak was observed in X-ray measurements of extrudates. It could be concluded that ibuprofen was molecularly dispersed in extrudates as an amorphous form.

### 3.3. Thermal Analysis

**Figure 5:**
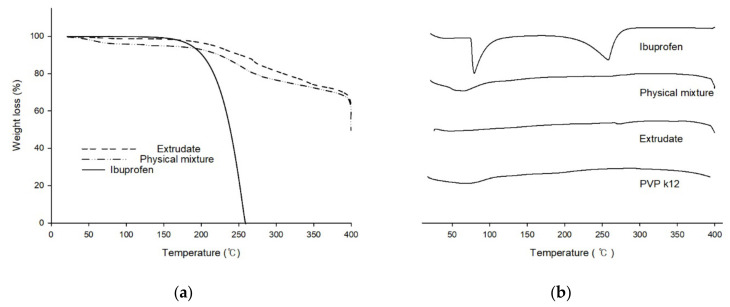
Thermal analysis result. (**a**) Thermogravimetry analysis (TGA) plots of extrudate, physical mixture and ibuprofen. (**b**) Differential thermal analysis (DTA) plot of ibuprofen, physical mixture, extrudate, and PVP K12.

To investigate the effect of 3D printing process on thermodynamic characteristics, thermogravimetric analysis (TGA) and differential thermal analysis (DTA) were performed. TGA and DTA results are shown in Figure 5. Evaporation of ibuprofen have been reported to start at approximately 158–190 °C and ended at approximately 245.7–277 °C [30,31,32]. As shown in Figure 5a, due to evaporation, significant weight loss of ibuprofen was observed at temperature over 180 °C. Approximately 80% of mass loss was observed near 250 °C. However, weight loss of physical mixture and extrudate was not observed to start over 250 °C. In DTA data, melting and boiling events of ibuprofen were observed at temperatures near 80 °C and 245 °C, respectively. However, these two endothermic events were not observed in results of physical mixture or extrudate.

### 3.4. Dose Adjustment

**Figure 6:**
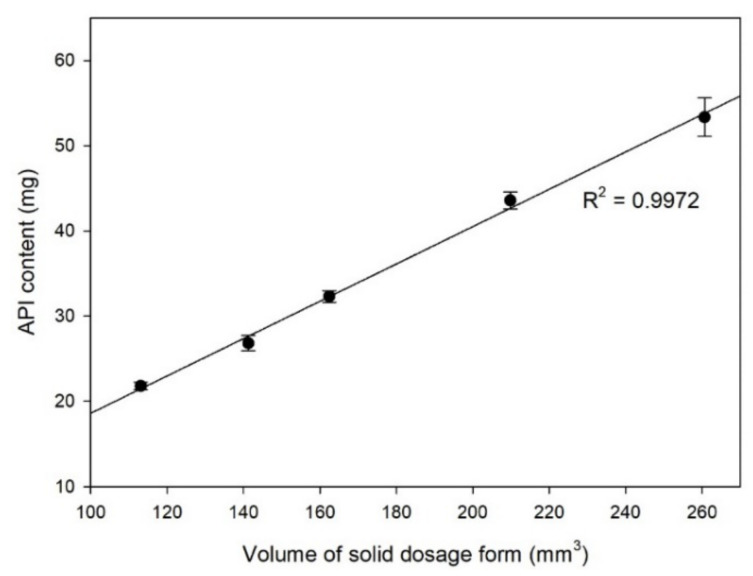
Correlation between the volume and API content of solid dosage form.

To demonstrate the applicability of HME 3D printer for personalization of doses for pharmaceutical dosage forms, the volume of ring dosage form was adjusted and then the API content of printed solid dosage form was analyzed. Figure 6 shows the changes of API content when the volume of solid dosage form was gradually increased. There was a high linear correlation between the volume of solid dosage form and the API content. This result indicates that a HME 3D printer has high flexibility and accuracy for adjusting doses of pharmaceutical dosage forms.

### 3.5. Floating Test

**Figure 7:**
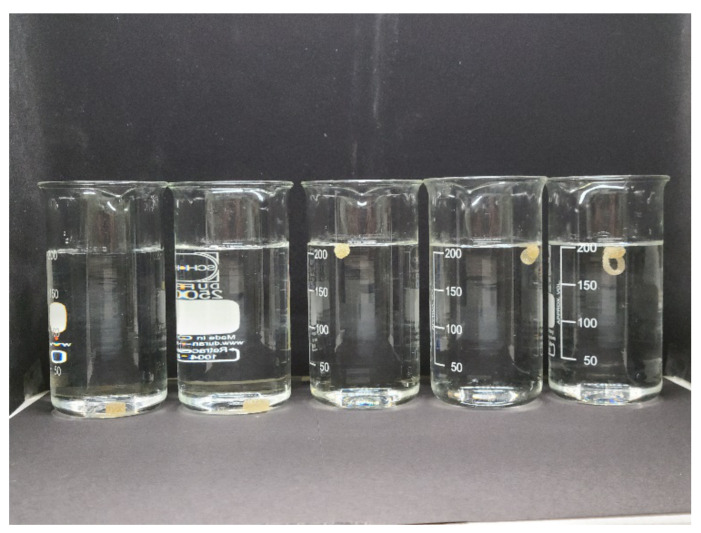
Floating comparison of cylinder, D12, float, cylinder–float (CF) and ring–float (RF)solid dosage forms from left.

As shown in Figure 7, floatable characteristics was successfully implanted to cylinder and D12 ring by fusion with floatable compartment. The apparent density of cylinder and D12 were over 1.00 g/cm^3^, and the true density of both solid dosage forms was about 1.16 g/cm^3^. By fusion with floatable compartment, the density of CF and RF were reduced under 0.90 g/cm^3^ (Table 1). Floating duration of float, CF, and RF were 22.5 ± 4.3 min, 14.4 ± 1.3 and 13.3 ± 1.7 min, respectively. This result indicates that it is possible to adjust behavioral characteristic of solid dosage form by combining with floatable compartment.

### 3.6. Dissolution Profile

#### 3.6.1. Dissolution Profile in Acidic Condition

**Figure 8:**
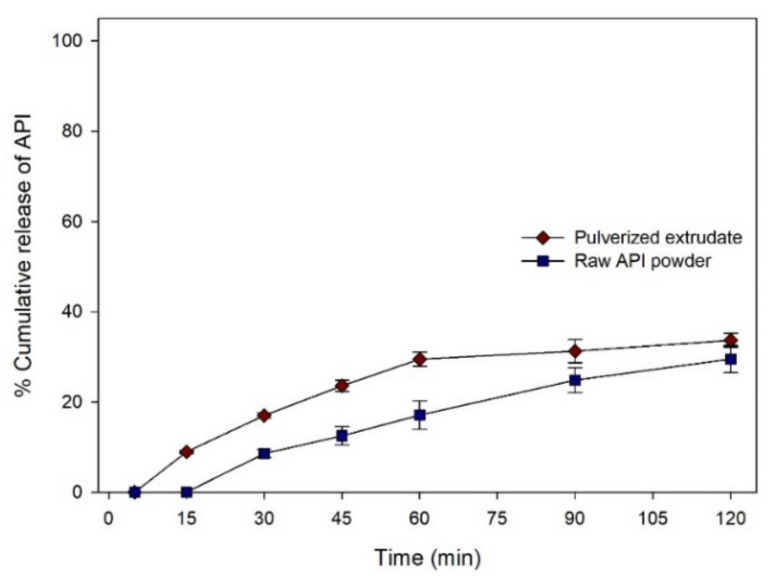
Dissolution rate of API powder and pulverized extrudate in pH 1.2 HCl solution.

Dissolution profiles of raw API powder (30 mg), and pulverized extrudate in pH 1.2 solution are plotted as shown in Figure 8. The sink condition was not maintained because of the low solubility of ibuprofen in acidic condition, and a low dissolution rate was observed under pH 1.2 condition. The percent API released from raw API powder and pulverized extrudate at 120 min was found to be 29.5 ± 3.0% and 33.7 ± 1.5% respectively. The dissolution rate of the pulverized extrudate was significantly higher than that of the raw API powder. (*p* < 0.05) Such enhancement of the dissolution rate might be because extrudates produced in the printing process were solid dispersions in which the API was present as amorphous form.

#### 3.6.2. Dissolution Profile in pH 6.8 Phosphate Buffer

**Figure 9:**
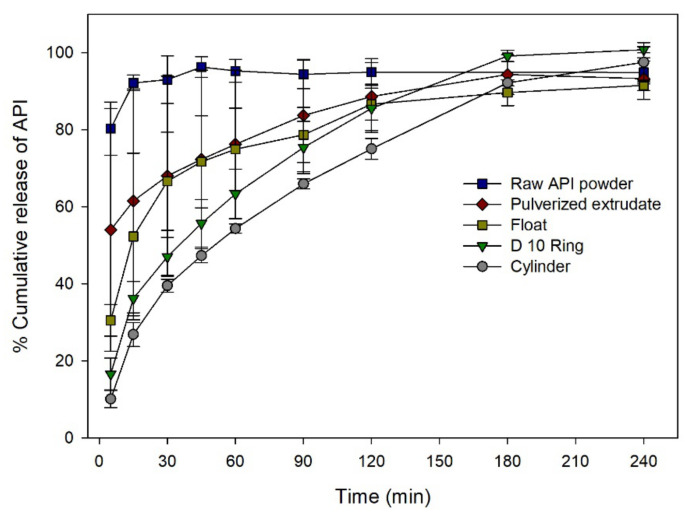
Dissolution rate of solid dosage forms in pH 6.8 phosphate buffer.

Dissolution profiles of raw API powder (30 mg), pulverized extrudate and solid dosage forms with various shapes in pH 6.8 solution were plotted. Results are shown in Figure 9. Because of the relatively high solubility of API, all solid dosage forms showed higher dissolution rates in pH 6.8 solution than in pH 1.2 condition. Raw API powder showed immediate API dissolution at an early phase of the test. Despite reduced crystallinity of API, pulverized extrudate and printed solid dosage forms showed sustained release due to polymeric hydrogel formation and the lack of a disintegrant. Depending on the geometrical properties, API release profiles were different. Pulverized extrudate showed faster dissolution rate than printed solid dosage forms due to the large surface area. For printed solid dosage forms, more than 80% of API release was observed within 120 min except cylinder solid dosage forms. Floatable solid dosage forms showed the fastest dissolution rate among printed solid dosage forms. On the other hand, cylinder solid dosage forms showed the slowest dissolution rate. Floatable solid dosage form could float on the surface of test medium at the early stage of test. After a few minutes, it adhered to the paddle or the paddle’s column. Floatable solid dosage forms were decomposed into small pieces very fast due to the stirring force. As a result, floatable solid dosage form showed the fastest dissolution rate among different models.

**Figure 10:**
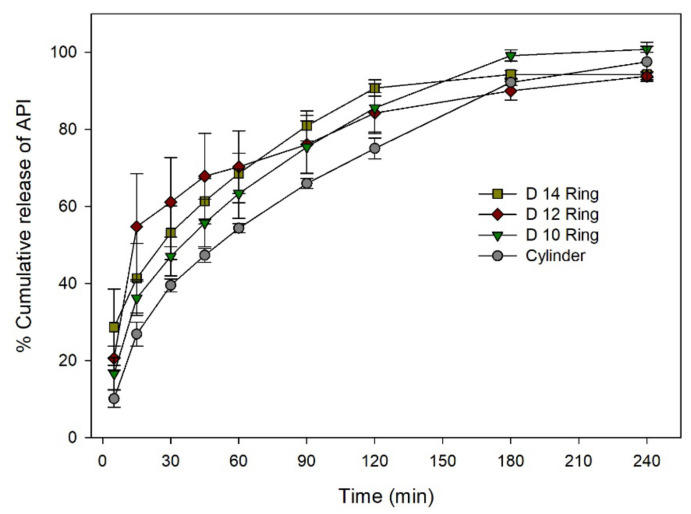
Dissolution rate of solid dosage forms in pH 6.8 phosphate buffer.

To better investigate the effect of SA/V on dissolution rate, two more ring solid dosage forms with different diameters and thicknesses were printed and evaluated. Figure 10 shows dissolution rates of three different ring solid dosage forms and cylinder solid dosage forms. Compared to cylinder, ring solid dosage forms showed relatively fast dissolution rates possibly due to their high SA/V values Ring solid dosage forms with a diameter of 14 mm (D14) showed fast dissolution rates. Because of the large surface area of D14, API started to dissolve fast in the initial stage of the test. However, D14 ring structure was limp and coiled to form large mass of hydrogel after a few minutes.

To compare the dissolution rate of solid dosage forms more clearly, % dissolution efficiency (% DE) of solid dosage forms at 5, 30, 90, and 180 min was calculated (Table 2). The % DE of cylinder solid dosage form was significantly lower than that of D10 ring at 5, 30, and 180 min (*p* < 0.05). The % DE of cylinder solid dosage form was significantly lower than that of D12 and D 14 ring at 5, 30, 90, and 180 min (*p* < 0.05).

For modification of dissolution rate or implantation of floatable character, solid dosage forms were fused in different combinations such as cylinder–cylinder (CC), cylinder–ring (CR), ring–ring (RR), cylinder–float (CF), and ring–float (RF). The D12 ring showed the fastest initial dissolution profile among ring solid dosage forms. It was applied for this fusion to observe changes of API release profiles more clearly.

**Figure 11:**
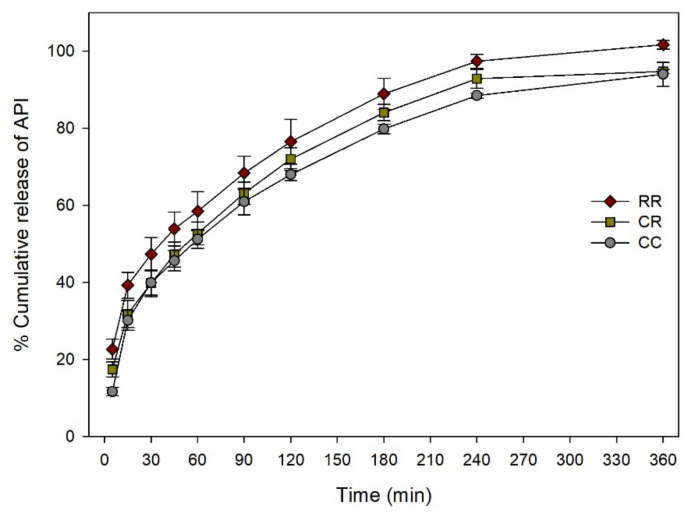
Dissolution rate change of non-floatable combined solid dosage forms.

Figure 11 shows dissolution rates of combined solid dosage forms that cannot float. SA/V ratios of CR and RR were 132% and 159% bigger than that of CC, respectively. Depending on the SA/V ratio, the dissolution rate was accelerated by replacing cylinder compartment to ring. More than 80% of API was dissolved within 180 min except for CC. Amounts of dissolved API of CR and RR at 5 min were 50% and 95% higher than that of CC, respectively. Dissolution rates of CR and RR at 180 min were 5%, 11% higher than that of CC, respectively. The % DE of fused solid dosage forms are shown in Table 2. The % DE of CC was significantly lower than that of CR at 5 and 180 min (*p* < 0.05). The % DE of CC was significantly lower than that of RR at 5, 30, 90, and 180 min (*p* < 0.05).

**Figure 12:**
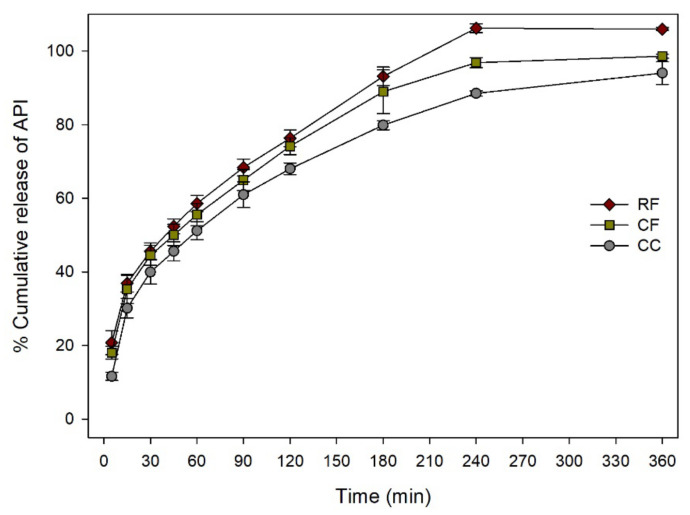
Dissolution rate of floatable combined solid dosage forms in pH 6.8 phosphate buffer.

Figure 12 shows dissolution rates of floatable combined solid dosage forms compared to CC. Although the SA/V was reduced by fusion of float solid dosage form, acceleration of dissolution rate was observed due to the floatable behavior. Amounts of dissolved API of CF and RF at 5 min were 56% and 78% higher than that of CC, respectively. Dissolution rates of CF and RF at 180 min were 11% and 17% higher than that of CC, respectively. The % DE of CC was statistically lower than that of CF at 5, 30, 90, and 180 min (*p* < 0.05). The % DE of CC was significantly lower than that of RF at 5, 30, 90, and 180 min (*p* < 0.05). These results indicated that fusion of solid dosage forms successfully modified the dissolution nature of solid dosage form. In the case of RR solid dosage form which had two D12 rings showed the highest dissolution rate. Because of the highest SA/V ratio, RR solid dosage form was dissolved very fast with a small size of hydrogel, resulting in a constantly fast dissolution rate.

## 4. Discussion

Combination of HME and FDM has been found to be useful for preparing both immediate release [17] and modified release [18] dosage forms. Hot-melt 3D extrusion process can be a novel candidate for pharmaceutical manufacturing. Because of a combined printing system of HME and FDM of 3D printer, the preparation step for filament is not required. Figure 1b shows the printing process of a hot-melt 3D extrusion. Hot-melt extrusion and 3-dimensional deposition of molten mixture take place using air compress barrel and nozzle in a single step. Moreover, low molecular or highly hydrophilic polymers, which form not suitable filament with low physical strength, flexibility and stability can be applied to HME 3D printer. Application of these polymers can accelerate API release for preparation of immediate-release dosage forms. HME has been used to prepare amorphous solid dispersion for increasing dissolution rate of poorly water-soluble APIs [33]. Because printing technology used in this study is based on HME, solid-state of API can be altered by a thermodynamic process. It can be applied for solubilization or enhancement of dissolution rate. Thus, HME 3D printing can simplify and reduce the process burden of pharmaceutical manufacturing in various aspects. Despite these advantages, the printer uses pneumatics, so using only a low viscosity polymer can be a disadvantage of this system. This weakness is thought to be solvable by the direct powder extrusion 3D printing recently reported by Goyanes et al. in 2019 [34], and more diverse studies on single-step processes should be conducted.

Application of hot-melt ram-extrusion 3D printing for the preparation of personalized orodispersible film has been reported [35]. In the present study, the feasibility of air driving HME based 3D printing for preparation of solid dosage form was evaluated. Thermodynamic condition might affect the solid-state of ibuprofen. Results of SEM, XRD, and DTA analyses revealed that API was dispersed in the polymeric matrix in an amorphous form (Figure 3, Figure 4 and Figure 5). Standard deviations of dose and weight of printed solid dosage forms were found to be very small, indicating that the printing process was stable to produce pharmaceutical dosage form with high uniformity. Significantly linear correlation between the volume of solid dosage form and API content was found, indicating that doses of dosage form could be personalized by adjusting model of solid dosage form.

Depending on characteristics of polymer and compactness of the solidified melt, printed solid dosage forms using FDM might show relatively slower dissolution rate than powder/granule compacted solid dosage forms [17]. Thus, not only solid-state of API, but also geometrical properties can affect the dissolution profile of solid dosage form. Ibuprofen has been reported to have pH-dependent solubility and show different dissolution profile depending on pH condition [36]. To understand effects of solid-state and geometrical properties on dissolution profile, dissolution test of printed solid dosage form was performed in HCl at pH 1.2 and phosphate solution at pH 6.8. The solid-state of API has a high effect on API dissolution profile. By using pH-dependent solubility of ibuprofen, the effect of thermodynamic condition of 3D printing process on dissolution profile that could be changed by solid-state of API was analyzed. Because of the low solubility of API in an acidic condition, a low dissolution rate was observed (Figure 8). Pulverized extrudate showed higher dissolution rate than raw API powder. Enhanced dissolution rate of pulverized extrudate might be caused by altered crystallinity of API (Figure 3, Figure 4 and Figure 5). Jain et al. [25] reported enhancement of solubility and dissolution rate of ibuprofen in solid dispersion systems using PEG 4000 and Tween 80. Thus, it is possible to prepare amorphous solid dispersion of API in water-soluble polymer matrix with hot-melt 3D extrusion.

It has been reported that dissolution profiles of 3D printed solid dosage forms can be different depending on their geometrical properties [37]. This indicates that not only solid-state of API, but also geometrical properties of solid dosage forms can play an important role in the dissolution profile (Figure 9). Moreover, it can also mean that geometrical properties can be more affective to the dissolution rate than solid-state of API in certain cases. Due to a relatively high solubility in phosphate solution at pH 6.8, dissolution rate was observed to be higher than that under an acidic condition. In contrast to the results in acidic condition, raw API powder showed a fastest dissolution rate in phosphate buffer at pH 6.8 (Figure 9). The relatively slow dissolution rate of pulverized extrudate might be due to polymeric hydrogel formation. Moreover, the disintegration time of solid dosage form might be delayed due to the lack of a disintegrant. The effect of geometrical properties on dissolution rate was observed distinctively under pH 6.8 condition due to a relatively high dissolution rate.

The dissolution rate was shown to be fast in order of float > ring > cylinder. Floatable behavior modified the movement of solid dosage forms more dynamically after solid dosage forms were introduced into the test medium. Dynamic movement makes solid dosage forms become split into small pieces of hydrogels, which accelerated the dissolution rate. Ring solid dosage forms showed faster dissolution rates than cylinder solid dosage forms due to their large surface area and characteristic shape with a hole in the center of solid dosage form. To understand effects of geometrical properties such as size and surface area more clearly, dissolution profiles of two more ring solid dosage forms with diameters of 12 mm (D12) and 14 mm (D14) were compared with those of D10 ring and cylinder solid dosage forms (Figure 10). It was found that ring solid dosage forms with high SA/V showed fast dissolution rates at the initial phase of dissolution. However, D14 ring solid dosage forms were limp and coiled to form large masses of hydrogels. This result indicates that, although surface area is essential for fast dissolution rate, physical strength for maintaining the structure during dissolving of solid dosage form is also essential. To improve physical strength, it is necessary to find a balance whether to manufacture in the shape of a conventional tablet or a new geometry for the controlled release of drugs. Improving and evaluating the physical strength of 3D printed solid dosage forms remains a challenge for the future.

As geometrical properties were found to be important for dissolution rate, the feasibility of using a new approach to modify the API release profile of dosage form was performed by combining different solid dosage forms together. Cylinder, D12 ring, and float solid dosage forms were fused in five combinations of CC, CF, RF, CR, and RR (Figure 2b). Floatable characteristic was implanted to a non-floatable solid dosage form by simple fusion with a floatable solid dosage form. Due to the floatable characteristic, solid dosage form dissolution was accelerated (Figure 12). By replacing compartment of cylinder solid dosage form with D12 ring compartment, it was possible to change SA/V ratio and result in a different dissolution rate (Figure 11). Application of 3D printer enables us to build a complex structure that cannot be prepared with current pharmaceutical manufacturing process. There are several approaches to produce solid dosage forms with different geometrical properties with a 3D printer [37]. However, modeling a dosage form with a specific API release profile can be a tricky task for local pharmacy or hospital. If the target dosage form has specific functionalized characteristics such as floatable or modified API release system, modeling could be more difficult. Fusion of solid dosage forms of specific dissolution profile can be an easy and fast method for modifying API release profile of dosage forms. The only thing the local pharmacy needs to do is to select prepared models and fuse them together, which is much simpler and faster way than modeling new solid dosage forms from the beginning.

## 5. Conclusions

In this study, the feasibility of air driving hot-melt 3D extrusion for preparing pharmaceutical dosage form was demonstrated. By using hot-melt 3D extrusion, solid dispersion of APIs can be deposited in an on-demand process without requiring a filament preparation step. Hot-melt 3D extrusion process showed high reproducibility and accuracy for preparing dosage forms. It was found that doses and release profiles of API can be customized by changing or combining 3D modeling. Higher solubility and faster dissolution rate of API can be achieved by altering API crystallinity and formation of solid dispersion. Given its high processability, hot-melt 3D extrusion is expected to be useful for preparing pharmaceutical dosage form for personalized medication with a simple and fast process. However, the limitation of available polymers is a challenge that needs to be addressed since it is a 3D printer using a pneumatic system. Furthermore, as heat is applied to the drug during the manufacturing process, it remains a undertaking in future to determine stability and self-life.

## Figures and Tables

**Table 1:** Physical properties of printed solid dosage forms. (*n* = 3, Mean ± S.D.)

Shape	Weight (mg)	Surface Area (mm^2^)	Volume (mm^3^)	SA/V	Apparent Density (g/cm^3^)
Cylinder	163.33 ± 0.49	188.11 ± 0.21	159.20 ± 0.44	1.18 ± 0.00	1.03 ± 0.00
D10 Ring	162.00 ± 3.99	263.57 ± 5.29	156.96 ± 3.87	1.68 ± 0.01	1.03 ± 0.00
D12 Ring	172.37 ± 3.86	322.65 ± 5.89	168.28 ± 3.77	1.92 ± 0.01	1.02 ± 0.00
D14 Ring	182.17 ± 1.96	377.20 ± 3.31	176.47 ± 1.90	2.14 ± 0.01	1.03 ± 0.00
Float	187.53 ± 3.13	227.16 ± 3.08	287.06 ± 5.84	0.79 ± 0.01	0.65 ± 0.00
CC	274.97 ± 2.61	311.05 ± 2.41	267.91 ± 2.54	1.16 ± 0.00	1.03 ± 0.00
CF	312.50 ± 10.50	345.05 ± 9.48	396.51 ± 16.32	0.87 ± 0.01	0.79 ± 0.00
CR	275.93 ± 8.75	411.60 ± 10.63	268.62 ± 8.52	1.53 ± 0.02	1.03 ± 0.00
RF	321.50 ± 1.51	470.34 ± 1.81	403.90 ± 2.33	1.16 ± 0.00	0.80 ± 0.00
RR	339.10 ± 7.01	603.56 ± 10.21	327.99 ± 6.78	1.84 ± 0.01	1.03 ± 0.00

**Table 2:** The % DE of dosage forms in pH 6.8 at 5, 30, 90, and 180 min. (*n* = 6, Mean ± S.D.)

Dosage Forms	% DE_5_	% DE_30_	% DE_90_	% DE_180_
Raw API powder	40.2 ± 3.2	81.7 ± 2.3	90.6 ± 2.3	92.7 ± 2.5
Pulverized extrudate	27.0 ± 14.4	56.1 ± 24.2	69.4 ± 20.1	79.6 ± 13.6
Cylinder	5.0 ± 1.0	23.6 ± 2.0	43.6 ± 1.5	61.4 ± 1.4
D 10 Ring	8.3 ± 1.9	31.0 ± 3.7	51.9 ± 5.0	70.2 ± 4.6
D 12 Ring	10.3 ± 1.4	43.2 ± 8.5	61.1 ± 8.7	72.9 ± 6.2
D 14 Ring	14.3 ± 4.5	37.7 ± 7.7	57.9 ± 5.4	74.1 ± 3.0
Float	15.3 ± 1.9	46.1 ± 11.4	64.7 ± 9.8	75.5 ± 6.0
CC	5.8 ± 0.5	25.4 ± 1.9	42.4 ± 2.2	56.6 ± 1.9
CR	8.7 ± 0.9	27.5 ± 2.5	44.1 ± 2.7	59.3 ± 2.5
RR	11.3 ± 1.2	33.8 ± 2.8	50.2 ± 3.6	64.8 ± 3.7
CF	9.1 ± 0.8	30.3 ± 2.3	46.9 ± 2.5	62.2 ± 2.0
RF	10.4 ± 1.5	32.0 ± 2.1	49.2 ± 1.9	64.9 ± 1.8.
